# Novel pulmonary perfusion imaging using chest digital dynamic radiography for pulmonary artery sarcoma

**DOI:** 10.1002/rcr2.737

**Published:** 2021-03-09

**Authors:** Shota Yamamoto, Fumio Sakamaki, Genki Takahashi, Ryotaro Yuji, Tomohiro Matsumoto, Terumitsu Hasebe

**Affiliations:** ^1^ Department of Radiology Tokai University Hachioji Hospital, Tokai University School of Medicine Tokyo Japan; ^2^ Department of Respiratory Medicine Tokai University Hachioji Hospital, Tokai University School of Medicine Tokyo Japan; ^3^ Department of Radiological Technology Tokai University Hachioji Hospital, Tokai University School of Medicine Tokyo Japan

**Keywords:** Digital dynamic radiography, perfusion–ventilation mismatch, pulmonary artery sarcoma, pulmonary perfusion, X‐ray

## Abstract

Chest digital dynamic radiography (DDR) is a novel method for evaluating pulmonary perfusion and ventilation. It could depict ventilation–perfusion mismatch in a pulmonary artery sarcoma with severe stenosis in the right pulmonary artery. This report is the first demonstration of ventilation–perfusion mismatch in a malignant neoplasm using DDR.

## Graphical abstract

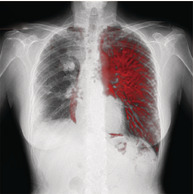

## Clinical Image

**Figure 1:**
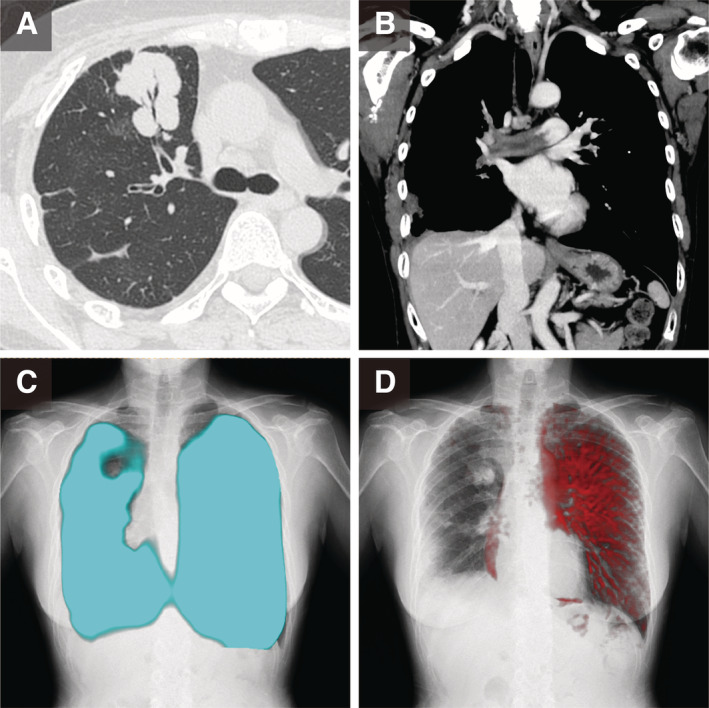
Chest imaging. (A) Chest computed tomography (CT) (lung window). The air bronchogram indicated three nodules (range: 14–26 mm) and interstitial thickening in the right upper lung lobe (S^3^). (B) Coronal contrast chest CT (mediastinal window). Severe stenosis in the right pulmonary artery was due to pulmonary arterial wall thickening and a low‐attenuated lesion in the right pulmonary artery. (C) A chest digital dynamic radiography (DDR) ventilation image showing pulmonary air filling during deep breathing for 15 sec, indicating almost normal bilateral lung ventilation dynamics. (D) A chest DDR perfusion image showing the blood supply while breath‐holding for 6 sec as slight changes in pixel value even without the use of contrast media. Severely decreased perfusion in the entire right lung can be observed.

An asymptomatic 51‐year‐old woman visited our hospital with an abnormal shadow observed during her medical check‐up. Chest computed tomography (CT) showed a tumorous shadow in the right lung field (Fig. 1A). CT pulmonary angiography (Fig. 1B) revealed severe stenosis in the right pulmonary artery due to arterial wall thickening and a low‐attenuated lesion. After CT‐guided biopsy, she was clinically diagnosed with pulmonary artery sarcoma and chemotherapy was initiated at another institute. Pulmonary perfusion and ventilation images were created from chest digital dynamic radiography (DDR), captured using a flat‐panel detector (AeroDR fine; Konica Minolta, Inc., Japan) and an X‐ray system (RADspeed Pro; Shimadzu Corporation, Japan) using KINOSIS 1.00 (Konica Minolta, Inc.). DDR technology is a novel evaluation of pulmonary perfusion and ventilation that provides a functional assessment in 6–15 sec with no contrast agent [1, 2]. It can be performed in standing or supine position [2] and requires less radiation exposure than scintigraphy. These images showed normal bilateral lung ventilation (Fig. 1C, Video S1) and severely decreased perfusion in the right lung (Fig. 1D, Video S2). This is the first demonstration of pulmonary ventilation–perfusion mismatch in pulmonary artery sarcoma using DDR. DDR would be beneficial in screening and follow‐up for patients with pulmonary perfusion abnormalities.

### Disclosure Statement

Appropriate written informed consent was obtained for publication of this case report and accompanying images.

### Author Contribution Statement

Fumio Sakamaki, Genki Takahashi and Tomohiro Matsumoto recruited the participants of this study. Shota Yamamoto wrote the primary draft of this manuscript. Ryotaro Yuji took clinical images and wrote on radiological techniques. Terumitsu Hasebe reviewed the final manuscript. All authors read and approved the final manuscript.

## Supporting information


**Video S1.** Chest digital dynamic radiography for ventilation.

**Video S2.** Chest digital dynamic radiography for perfusion.
